# Measuring sugar intake in oral health birth cohort studies: a scoping review

**DOI:** 10.3389/fnut.2025.1667487

**Published:** 2026-01-07

**Authors:** Shilpa Sarawagi, Karla Gambetta-Tessini, Silas Alves-Costa, Marly Augusto Cardoso, Gustavo G. Nascimento, Karen G. Peres

**Affiliations:** 1 National Dental Research Institute Singapore, National Dental Centre Singapore, Singapore, Singapore; 2 Oral Health Academic Clinical Programme, Duke-NUS Medical School, Singapore, Singapore; 3 Graduate Program in Dentistry, Federal University of Maranhão, São Luís, Brazil; 4 Department of Nutrition, School of Public Health, University of São Paulo, São Paulo, Brazil

**Keywords:** dietary assessment, sugar intake, sugar-sweetened beverage, birth cohort studies, oral health outcomes

## Abstract

Numerous reviews have explored the relationship between sugar intake and various health conditions, with specific recommendations for sugar intake thresholds in medical and dental research. However, heterogeneity in dietary assessment methods used to measure sugar intake has posed challenges. This scoping review aimed to identify and map the methods assessing sugar intake in oral health birth cohort studies (OHBCS). Using the Population–Concept–Context framework, the review included participants in OHBCS, dietary assessment methods measuring sugar intake associated with oral health outcomes from studies conducted worldwide. Data from PubMed, Embase, Web of Science, Scopus, and Dentistry & Oral Sciences Source were searched until June 2025, with no date and language restrictions. Articles from a previous OHBCS scoping review and its 3-year update were also included. From 2,297 screened articles, 34 studies representing 24 OHBCS across 13 countries (77% from high-income countries) met the inclusion criteria. Dietary assessment methods to assess sugar intake identified included Food Frequency Questionnaire (FFQ, *n* = 11), non-specific questionnaires (*n* = 9), food lists (n = 7), food diary (*n* = 7), 24-h recall (*n* = 5), and structured interviews (*n* = 1). Methods of estimating sugar intake varied, with frequency (*n* = 19) being the most common, followed by quantity (*n* = 11), number of sugar items introduced (*n* = 9), energy (*n* = 3), and the percentage of sugar consumption (*n* = 2). Different types of dietary sugars were assessed, including intrinsic sugars (*n* = 5), milk sugars (*n* = 14), and free sugars (*n* = 34). FFQs emerged as the most frequently used sugar intake assessment method, with a focus on intake frequency. This review highlights a significant lack of standardization in sugar intake assessment across studies, underscoring the need for a unified approach to guide early interventions, inform dietary recommendations, and enhance comparability in future OHBCS research.

## 1 Introduction

The World Health Organization (WHO) Global Oral Health Status Report (2022) stated that 45% of the global population suffers from one or more untreated oral diseases, surpassing other non-communicable diseases (NCDs) in prevalence (1). Oral diseases such as untreated dental caries, oral cancer, and periodontitis are urgent public health challenges with significant social, economic, and environmental consequences (2). According to the Global Burden of Disease Study 2021, untreated dental caries in permanent teeth is the most prevalent oral health condition worldwide, affecting 29.4% of the population—over 2 billion people. In deciduous teeth, untreated dental caries impacts 524 million children globally (3).

According to the WHO, the term “total sugars” includes intrinsic sugars (IS), which are those incorporated within the structure of intact fruit and vegetables; sugars from milk (lactose and galactose); and free sugars, which are monosaccharides and disaccharides added to foods and beverages by the manufacturer, cook or consumer, and sugars naturally present in honey, syrups, fruit juices, and fruit juice concentrates (4, 5). Added sugars include sugars added to food during food processing, sugars used as sweeteners, and sugars from honey and concentrated fruit or vegetable juices. Added sugars do not include naturally occurring sugars, such as sugars in the intact cell walls of fruit and vegetables or in milk (4). Caries lesions are caused by acids produced when free sugars are fermented by cariogenic biofilm (4). Free sugars in the diet are a common risk factor for dental caries and other NCDs, including cardiovascular diseases, obesity, and diabetes (6). In response, the WHO recommends limiting free sugar intake to less than 10% of total energy consumption, with further reductions to less than 5% for minimizing the risk of dental caries and obesity across the life course (4). Introducing sugars into a child’s diet during their first year of life significantly increases the risk for dental caries and obesity over their lifetime (7, 8).

Birth cohort studies offer the strongest scientific framework for examining the long-term relationship between sugar consumption and NCDs, including dental caries. These studies enable the assessment of causality and the natural history of diseases (9, 10). Oral Health Birth Cohort Studies (OHBCS) worldwide have routinely collected data on intake of sugars during early life (11), facilitating analyses of the temporal relationship between this exposure and development of caries from childhood (12) to adulthood (13–15). Identifying valid and reliable dietary assessment methods is critical for accurately studying the relationship between sugar intake and oral health. While poorly designed tools can obscure true associations (16), standardized dietary assessment methods improve the consistency and comparability of research findings, pivotal for evidence-based policymaking (17). Nonetheless, excessive data collection can increase costs, extend research timelines, and reduce participation rates.

Standardizing dietary assessment methods is imperative for global research harmonization. It enables location-specific public policy development, facilitates pooled analyses, and supports new OHBCS in adopting effective data collection practices. This study aimed to provide an overview of the methods used to assess sugar intake associated with oral health outcomes in OHBCS. The objective of this review was to identify and describe the dietary assessment methods employed in OHBCS.

## 2 Methods

The goal of scoping reviews is to rapidly map the key concepts underpinning a research area and the main sources and types of evidence available (18). This study followed the methodological framework for scoping reviews (18), including the five steps: (1) identifying the review questions, (2) identifying relevant studies, (3) selecting the studies, (4) charting the data, and (5) collating, summarizing, and reporting the results.

### 2.1 Review question

The review question was formulated using the Population, Concept, Context (PCC) strategy: the Population encompassed all participants enrolled in OHBCS; the Concept focused on assessment of sugar intake in relation to oral health outcomes, while the Context was specific to OHBCS worldwide. This approach led to the following review question:

“*What dietary assessment methods are used to measure sugar intake associated with oral health outcomes in OHBCS?”*

### 2.2 Search Strategy

The PRISMA extension for Scoping Reviews (PRISMA-ScR) (19) was followed for preferred reporting items. To identify OHBCS that investigated sugar intake and its association with oral health outcomes, one of the authors (SS) searched the electronic databases PubMed, Embase, Web of Science, Scopus, and Dentistry & Oral Sciences Source in January 2025 and updated the search on 17 June 2025, without language and publication date restrictions. A search strategy was developed using the search strings related to the following terms: (1) birth cohort studies, (2) oral health outcomes, and (3) sugars and dietary intake (details are given in Supplementary Table 1). Also, two authors (SS and SAC) searched the list of articles from OHBCS, which were mapped in a published scoping review (20) and its subsequent 3-year update (data under preparation). Additionally, the reference list of the selected articles was also hand-searched for relevant studies.

### 2.3 Eligibility criteria

Studies that met the following criteria were eligible for this scoping review: (1) studies with sugar intake as the main exposure, regardless of the method for data collection in OHBCS; (2) studies with any oral health outcome (self-reported or clinically diagnosed) associated with sugar intake; and (3) those with full text available. No language restriction was applied to this scoping review. Articles in languages other than English were translated using Google Translate, or researchers proficient in the language assisted in reviewing the articles. Exclusion criteria included: (1) studies that had sugars as a confounder/mediator; (2) studies presenting research proposals only and limiting the introduction of sugar-sweetened beverages by modifying environments or policies with no collected sugar intake information; (3) studies restricted to premature/low birth weight/high birth weight children or pregnant mothers; and (4) studies other than OHBCS.

### 2.4 Study selection

Articles identified in the electronic search were deduplicated using the EndNote 20 Version (21) and Rayyan software (22). Two trained and calibrated reviewers (SS and SAC) screened titles and abstracts independently. The first hundred articles in alphabetical order were used for training and calibration (Kappa interrater = 0.80; Agreement = 93.7%). Full texts of selected articles were then retrieved and examined for suitability for this study. Two reviewers were trained and calibrated for full-text reading, with the first ten articles in alphabetical order. Both reviewers read all of the selected articles independently. Any disagreements regarding the selection of studies were resolved through a discussion with a third reviewer (KGT) and two experts (GGN and KGP). Authors/researchers were contacted to retrieve the full text of unavailable articles or clarify methodological aspects of the articles.

### 2.5 Charting the data

Data were extracted from the articles into forms containing the following information: (1) OHBCS characteristics (name of the study, country, year of the study and sample size); (2) age of exposure and age at which the outcome was measured; (3) oral health outcomes; (4) statistical approach; (5) confounders; (6) main findings; (7) funding sources; and (8) dietary assessment methods, comprising the sugar intake assessment methods used in the selected studies, including the validation method reported in the article. Additionally, information on the respondents for dietary assessment methods was included. Sugar intake measurement was categorized into frequency (times/day or times/week or times/month or times/year), quantity (grams or ounces or milliliters), energy (caloric energy from sugars/day or total calorie intake/day), percentage (proportion of daily added sugar intake and density of sugars in percentage), and the number of sugary food items. All food items identified in the selected articles were systematically charted in the table.

### 2.6 Collating, summarizing, and reporting the results

Based on the food items described in the articles, the types of sugars measured were summarized into categories: free sugars (including added sugars), intrinsic sugars, and milk sugars (5). Additionally, the types of sugar intake assessment methods employed in the studies were reported. OHBCS included in this review were depicted on a world map illustrating the number of articles from each cohort.

## 3 Results

**Figure 1:**
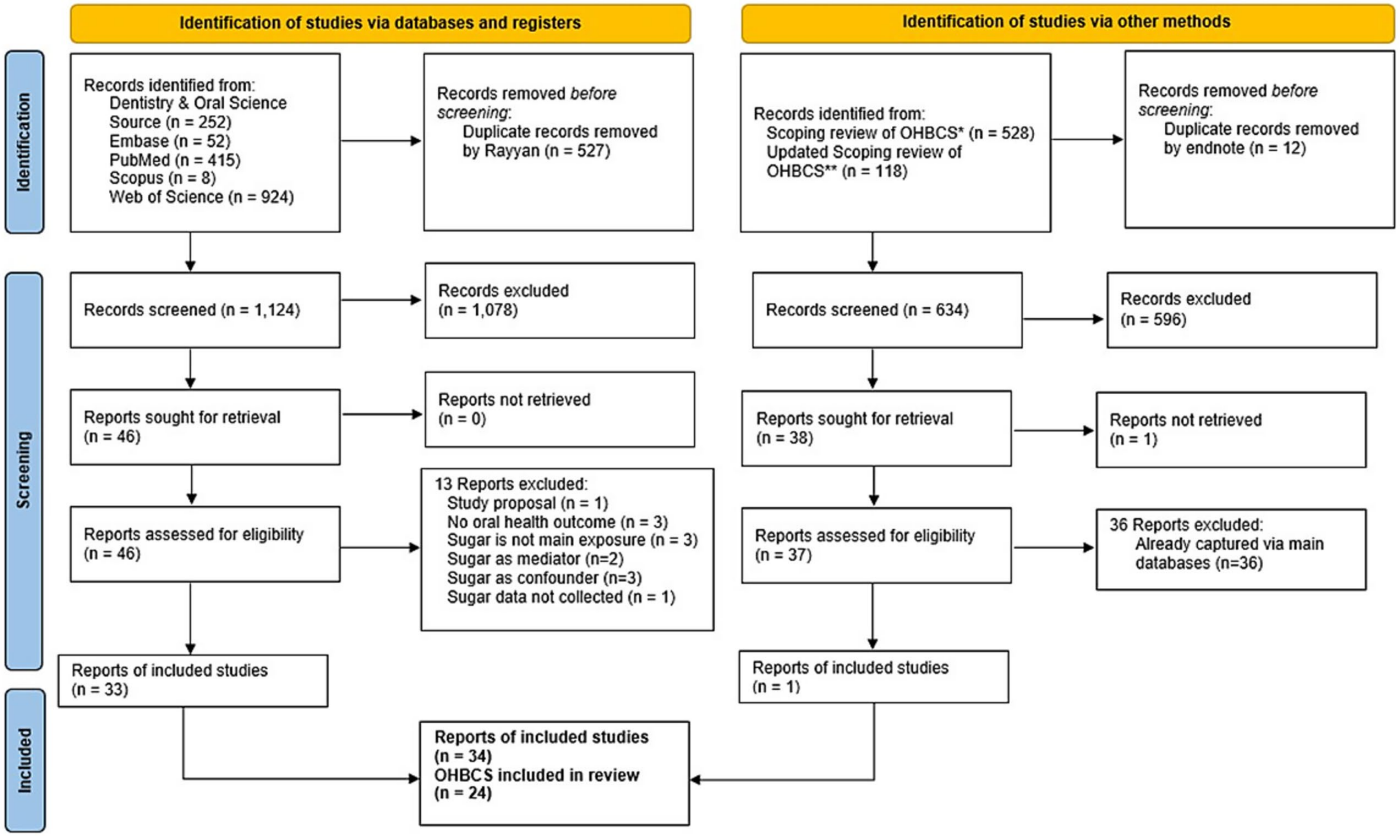
PRISMA flowchart for selection of articles. *Peres et al. (20). **Updated scoping review 2025 (data under preparation).

Figure 1 depicts the PRISMA flow for the identification and selection of studies. The database search identified 1,651 articles, of which 527 were duplicates. Thus, 1,124 articles had their title and abstracts screened. After this stage, 46 articles were deemed relevant for full-text reading, of which 33 articles met the eligibility criteria and were included. Additionally, 646 articles were screened from the 2022 scoping review of OHBCS (20) and its updated version in 2025, adding a total of 2,297 articles for title and abstract screening. One article out of these 646 articles was considered eligible for this review. In total, 34 articles were included for data extraction from 24 OHBCS (7, 8, 11–13, 23–51). Full details of the excluded articles are reported in Supplementary Table 2.

### 3.1 Characteristics of selected articles

**Figure 2:**
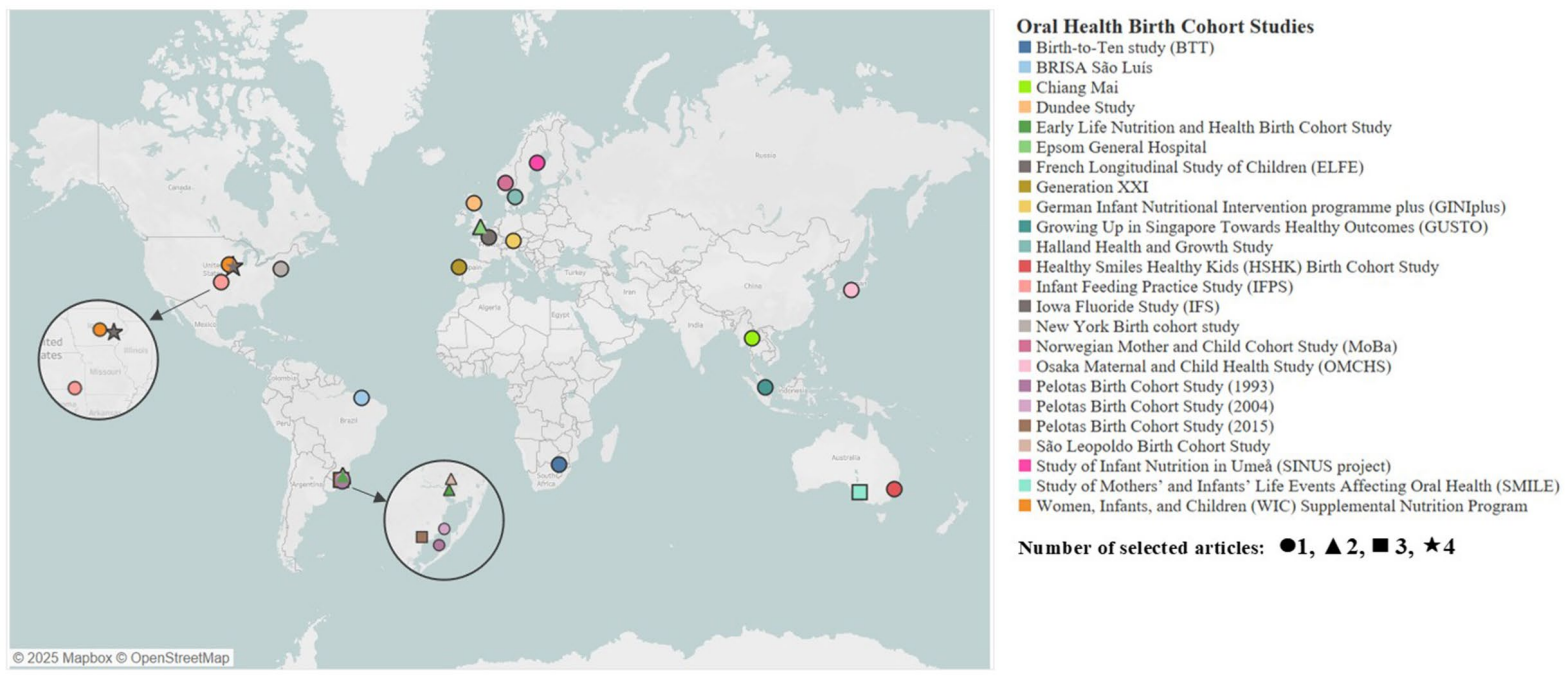
Geographical distribution of selected OHBCS.

The 34 included articles were published from 2000 to 2025, representing 24 OHBCS conducted in 13 countries worldwide. Among these countries, high-income countries comprised the majority (*n* = 10, 77%), followed by upper-middle-income countries (*n* = 3, 23%). Most studies were conducted in Brazil (OHBCS = 6, articles = 10) (7, 11, 28, 34, 39, 41, 43, 46, 47, 50), the United States of America (OHBCS = 4, articles = 7) (12, 13, 26, 30, 32, 33, 49), Australia (OHBCS = 2, articles = 4) (35, 40, 44, 48), the United Kingdom (OHBCS = 2, articles = 3) (8, 24, 25), and Sweden (OHBCS = 2, articles = 2) (27, 42). France (51), Germany (37), Japan (29), Norway (31), Portugal (38), Singapore (36), South Africa (23), and Thailand (45) contributed to one study from one OHBCS each (Figure 2).

The sample size for sugar data collection in the selected articles ranged from 86 to 10,921 participants. The age range for sugar exposure was 2 months to 18–19 years, whereas the age range for oral health outcomes was 1 year to 18–19 years. Dental caries was the most commonly investigated oral health outcome related to intake of sugars (*n* = 28 articles); however, the methods for reporting dental caries varied widely, including the DMF index (number of decayed, missing, filled teeth or surfaces), International Caries Detection and Assessment System (ICDAS) criteria, presence of cavitated/non-cavitated caries lesions, severe early childhood caries, prevalence of caries, and self-reported dental caries (7, 8, 11–13, 23, 27–31, 33–40, 42–48, 50, 51). Other oral health outcomes related to intake of sugars included the microbiome (*S. mutans* and *Candida*) in plaque/saliva (*n* = 3) (25, 32, 49), dental plaque (*n* = 1) (24), tooth wear (*n* = 1) (26), and periodontal disease (*n* = 1) (41) (Table 1).

**Table 1:** Main characteristics of the selected articles.

No	Author/Year	Country	Cohort’s name	Sample size (n)	Age/age range (Exposure)	Age/age range (outcome)	Oral Health Outcome (s) associated with sugar
1	MacKeown et al. 2000 (23)	South Africa	Birth-to-Ten Study (BTT)	259 (longitudinal; 1y and 5y both) 1,216 (cross-sectional; 1y) 164 (cross-sectional; 5y)	1 and 5 y	1 and 5 y	Dental caries (dmfs)
2	Habibian et al. 2001 (24)	United Kingdom	Epsom General Hospital	163	6, 12, and 18 mo	12 and 18 mo	Visible dental plaque
3	Habibian et al. 2002 (25)	United Kingdom	Epsom General Hospital	163	6, 12, and 18 mo	12 and 18 mo	*S. mutans* in the plaque
4	Warren et al. 2002 (26)	United States of America (USA)	Iowa Fluoride Study (IFS)	355	6, 9, 12, 16, 20, 24, 28, 32, 36, 42, and 48 mo	4.5 to 5 y	Tooth wear
5	Marshall et al. 2003 (12)	United States of America (USA)	Iowa Fluoride Study (IFS)	636 (1 y), 525 (2 y), 441 (3 y), 410 (4 y), 417 (5 y), and 396 (cumulatively for 1 through 5 y)	1, 2, 3, 4, and 5 y	4 to 7 y	Dental caries (dfs, dft included cavitated and non-cavitated lesions)
6	Öhlund et al. 2007 (27)	Sweden	Study of Infant Nutrition in Umeå (SINUS project)	86	12 mo	4 y	Dental caries (dmfs, dfs)
7	Feldens et al. 2010 (28)	Brazil	São Leopoldo Birth Cohort Study	340	6 and 12 mo	4 y	Severe early childhood caries (S-ECC)
8	Tanaka et al. 2013 (29)	Japan	Osaka Maternal and Child Health Study (OMCHS)	315	2 to 9, 16 to 24, 29 to 39, and 41 to 49 mo	41 to 50 mo	Dental caries (dft), Early Childhood Caries (ECC)
9	Chaffee et al. 2015 (7)	Brazil	Early Life Nutrition and Health Birth Cohort Study	458	6 and 12 mo	38 mo	Dental caries (dmft including non-cavitated lesions) Severe early childhood caries (S-ECC)
10	Park et al. 2015 (30)	United States of America (USA)	Infant Feeding Practice Study (IFPS)	1,274	6, 10, and 12 mo, and 6 y	6 y	Number of dental cavities (reported by the parents)
11	Wigen and Wang 2015 (31)	Norway	Norwegian Mother and Child Cohort Study (MoBa)	1,095	1.5 and 5 y	5 y	Dental caries or dmft = 0
12	Peres et al. 2016 (11)	Brazil	Pelotas Birth Cohort Study (1993)	359 at 6 y, 339 at 12 y, 307 at 18 y	4, 15, and 18 y	6, 12, and 18 y	Dental caries (dmft and DMFT)
13	Avasare et al. 2017 (32)	United States of America (USA)	Women, Infants, and Children (WIC) Supplemental Nutrition Program	94	Baseline: 6–24 months, Baseline +9 months, Baseline +18 months	Baseline, 9 mo and 18 mo	*S. mutans* in saliva
14	VanBuren et al. 2017 (33)	United States of America (USA)	Iowa Fluoride Study (IFS)	344	from 9 to 17 year (semi-annually)	5, 9, 13, and 17 y	Dental caries (DFS) into Clusters: 1 = “low DFS,” 2 = “medium DFS,” 3 = “high DFS”
15	Feldens et al. 2018 (34)	Brazil	Early Life Nutrition and Health Birth Cohort Study	345	12 mo	38 mo	Dental caries (d_1_mft, d_1_mfs); (ECC and S-ECC)
16	Bell et al. 2019 (35)	Australia	Study of Mothers’ and Infants’ Life Events Affecting Oral Health (SMILE)	680	12 mo	24–36 mo	Dental Caries (ICDAS, dmfs) and (ECC)
17	Hu, Shijia et al. 2019 (36)	Singapore	Growing Up in Singapore Towards Healthy Outcomes (GUSTO)	363	6, 9 and 12 mo	2 and 3 y	ECC (modified ICDAS)
18	Bernabé et al. 2020 (8)	United Kingdom (UK)	Dundee Study	1,111	12–48 mo	12–48 mo	ECC (dmfs)
19	Pitchika et al. 2020 (37)	Germany	German Infant Nutritional Intervention Programme Plus (GINIplus)	915 (10 y) and 996 (15 y)	10 and 15 y	10 and 15 y	Dental Caries (non-cavitated caries lesions, dmfs)
20	Carvalho Silva et al. 2021 (38)	Portugal	Generation XXI	607	4 y	4 and 7 y	Dental caries (d_3–6_mft/D_3–6_MFT; ICDAS)
21	Feldens et al. 2021 (39)	Brazil	Early Life Nutrition and Health Birth Cohort Study	233	6 month (sugar consumption index), 3 year (household sugar purchases)	3 and 6 y	Dental caries (dmft, DMFT)
22	Manohar et al. 2021 (40)	Australia	Healthy Smiles Healthy Kids (HSHK) Birth Cohort Study	718	4 mo, 8 mo, 1 y, 2 y, and 3 y	3 and 4 y	ECC (dmft, dmfs)
23	Marshall et al. 2021 (13)	United States of America (USA)	Iowa Fluoride Study (IFS)	318	1–17 y	17 y	Dental caries (DFS)
24	Moreira et al. 2021 (41)	Brazil	São Luís	2,515	18–19 y	18–19 y	Periodontal disease: full mouth visible plaque index (FMPI) + Bleeding on probing (BoP) + Periodontal Probing Depth (PPD) + Clinical attachment level (CAL)
25	Boustedt et al. 2022 (42)	Sweden	Halland Health and Growth Study	208	6, 12, 18 and 24 month	5 year	ECC (dmft)
26	Echeverria et al. 2022 (43)	Brazil	Pelotas Birth Cohort Study (2015)	3,654	3, 12, 24, and 48 mo	48 mo	ECC (simplified ICDAS)
27	Ha et al. 2022 (44)	Australia	Study of Mothers’ and Infants’ Life Events Affecting Oral Health (SMILE)	879	birth-age to 5 y (Mothers’ sugar-sweetened beverages intake)	5 y	Dental caries (dmfs)
28	Wu, Tong et al. 2022 (45)	Thailand	Chiang Mai	350	3 and 5 y	3 and 5 y	ECC (dmft)
29	da Silva et al. 2023 (46)	Brazil	Pelotas Birth Cohort Study (2004)	996	10–11 y	12 and 13 y	Dental caries (DMFS)
30	Echeverria et al. 2023 (47)	Brazil	Pelotas Birth Cohort Study (2015)	3,654	3, 12, 24, and 48 mo	48 mo	ECC (simplified ICDAS)
31	Ha et al. 2023 (48)	Australia	Study of Mothers’ and Infants’ Life Events Affecting Oral Health (SMILE)	879	1 y, 2 y, and 5 y	5 y	Dental caries (dmfs)
32	Alkadi et al. 2024 (49)	United States of America (USA)	New York Birth Cohort Study	160	12, 18 and 24 mo	12, 18 and 24 mo	*S. mutans* and *Candida* in saliva/plaque
33	Mathias et al. 2024 (50)	Brazil	Pelotas Birth Cohort Study (2015)	3,645	24 mo	48 mo	Dental caries (ICDAS and dmfs) and ECC
34	Kerguen et al. 2025 (51)	France	French Longitudinal Study of Children	10,921	2 to 6 mo	3.5 y	ECC (reported by the parents)

ECC, Early Childhood Caries; S-ECC, Severe Early Childhood Caries.

### 3.2 Types of sugar intake assessment methods and their characteristics

**Figure 3:**
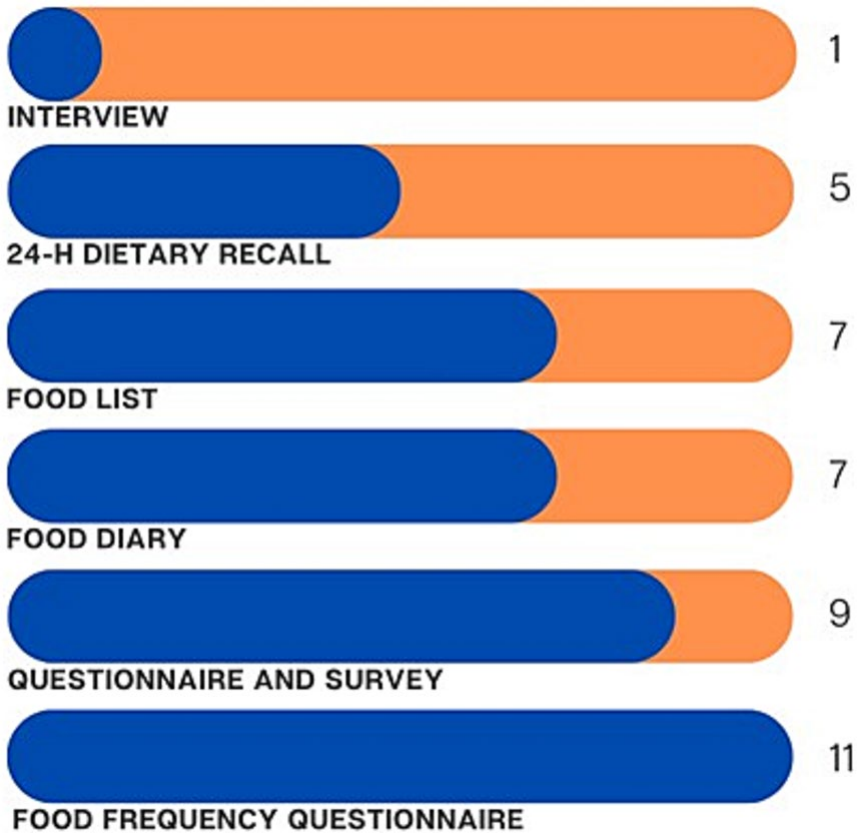
Sugar intake assessment methods reported in selected articles **numbers do not sum up to the total number of selected articles due to more than one instrument being reported.

**Figure 4:**
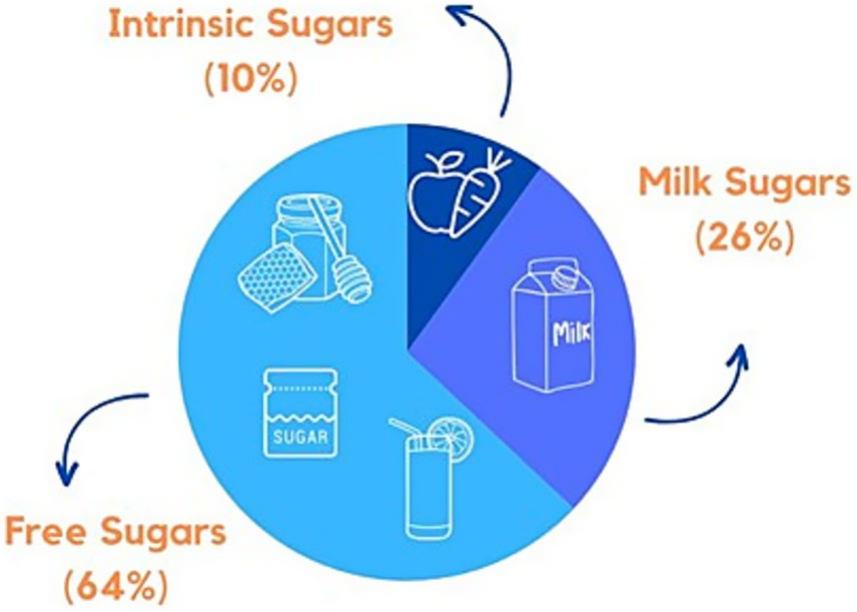
Proportion of articles according to classification of dietary sugars.

Various methods were used to measure sugar intake in the studies, with Food Frequency Questionnaires (FFQ) being the most common (*n* = 11) (11, 13, 23, 33, 37, 38, 40, 41, 46, 48, 51), followed by non-specific questionnaires (*n* = 9) (8, 26, 29–32, 42, 45, 49), food lists (checklist of pre-defined food items) (*n* = 7) (7, 11, 34, 43, 44, 47, 50), food diaries (*n* = 7) (12, 24, 25, 27, 35, 36, 48), 24-h recall (*n* = 5) (28, 34–36, 48), and structured interviews (*n* = 1) (28) (Figure 3). Six articles utilized FFQs to estimate sugar intake quantities (13, 23, 33, 37, 46, 48), while five used these instruments to report the daily frequency of sugar intake (11, 38, 40, 46, 51). There were only three articles that reported both frequency and quantity of sugar intake per day (27, 36, 46). Only 13 articles reported the validation or piloting of these assessment methods (13, 23–25, 28, 32, 37, 38, 41, 46–48, 51), and two mentioned adapting them from previous studies (40, 49). The primary methods for estimating sugar intake included frequency (*n* = 19) (8, 11, 24–31, 34, 36, 38, 40, 42, 44–46, 51), quantity (*n* = 11) (12, 13, 23, 27, 32, 33, 36, 37, 39, 46, 48), number of sugary items introduced (*n* = 9) (7, 11, 28, 39, 43, 44, 47, 49, 50), energy (*n* = 3) (35, 37, 46), and percentage of sugar consumption (*n* = 2) (28, 41) (Table 2). Retrospective methods of assessment were commonly used (*n* = 27) (7, 8, 11, 13, 23, 26, 28–34, 37–47, 49–51) while prospective methods were reported in only four articles (12, 24, 25, 27). Three articles reported both prospective and retrospective assessment methods for estimating sugar intake (35, 36, 48). No study reported digital devices for collecting dietary information on participants’ diets. The high-sugar food items studied in the articles are detailed in Table 2. The articles examined different types of dietary sugars: intrinsic sugars (*n* = 5) (7, 23, 35, 48, 49), milk sugars (*n* = 14) (7, 12, 13, 23–25, 32–35, 40, 46, 48, 49), and free sugars (*n* = 34) (7, 8, 11–13, 23–51) (Figure 4). Most OHBCS found a positive association between high sugar intake and dental caries, with early exposure in infants associated with an increased caries experience later in life (*n* = 21) (7, 8, 11–13, 23, 28–31, 33, 34, 37, 39, 42, 43, 45–48, 50, 51). However, a few articles (*n* = 5) (27, 35, 36, 38, 40) did not confirm this association. Some OHBCS also indicated that high sugar intake could increase levels of salivary and plaque *S. mutans* and *Candida* (*n* = 3) (25, 32, 49); however, one study did not find a significant association between visible plaque and frequency of high sugar intake (24). Maternal consumption of SSB during pregnancy and the early postnatal period was associated with dental caries in their children (*n* = 1) (44). Toothwear was not associated with dietary sugar intake (*n* = 1) (26), whereas added sugar consumption was found to be associated with a higher risk of periodontal disease in adolescents (*n* = 1) (41). Various statistical analyses were employed in OHBCS (details reported in Supplementary Table 3). Covariates adjusted for in the statistical analysis are presented in Supplementary Table 4.

**Table 2:** Characteristics of dietary assessment methods and the main exposure (sugar intake).

N°	Author/Year	Dietary assessment methods	Measurement of sugar (quantity, frequency, percentage, energy, and number of sugary food items)	Respondent (self-administered or researcher-led/interviewer-administered) and reference food intake period	Type of sugars and food items (Intrinsic sugars (IS), milk sugars (MS), free sugars (FS) include naturally occurring sugars and added sugars)
1	MacKeown et al. 2000 (23)	Semiquantitative Food Frequency Questionnaire (FFQ)^1^ (65 items)	Quantity (g) (mean daily intake)	Parents or guardians (asked by multilingual trained interviewers)	IS, MS, FS: Six food groups: (1) Grain/cereal group, breakfast cereal/porridges and other starches, (2) Meat and meat substitutes, (3) Fruit and vegetable, (4) Fats and oils, (5) Milk and milk products, (6) Miscellaneous (contains sugars) (67)
2	Habibian et al. 2001 (24)	3-day food diary^1^	Frequency (times/day)	Parents or carers (self-administered) (intake in 3 days, including 2 weekdays and 1 weekend)	MS, FS: Cake, Chocolates Crisps, Sweets, Sugared cereals Fruit, Sugared milk Cow’s milk, Formula milk, Soya milk, Formula milk with Non-Milk Extrinsic Sugars (NMES): Sugared drinks, Sugared beverages, Fruit juice, Water, Drinks no sugars Milk desserts, non-milk desserts.
3	Habibian et al. 2002 (25)	3-day food diary^1^	Frequency (times/day)	Parents or carers (self-administered) (intake in 3 days, including 2 weekdays and 1 weekend)	MS, FS: Cake, Chocolates Crisps, Sweets, Sugared cereals Fruit, Sugared milk Cow’s milk, Formula milk, Soya milk, Formula milk with Non-Milk Extrinsic Sugars (NMES): Sugared drinks, Sugared beverages, Fruit juice, Water, Drinks no sugars Milk desserts, non-milk desserts.
4	Warren et al. 2002 (26)	Questionnaire	Frequency (number of occasions per day)	Parents (self-administered)	FS: Juices and juice drinks, soft drinks, and sweetened powdered beverages mixed with water.
5	Marshall et al. 2003 (12)	3-day food and beverage diaries	Quantity (g) (mean daily intake)	Parents (self-administered)	MS, FS: Dairy foods and all beverages were classified by food type (e.g., fluid milk, cheese, 100% juice, juice drink, water). For foods having cheese or milk as an ingredient (e.g., lasagna, cheeseburger), the dairy component was estimated from ingredient weights and included in the appropriate group. Non-milk dairy intake is the sum of cheese, yogurt, and dairy desserts. Total sugared beverage intake is the sum of 100% juice, juice drinks, regular (i.e., sweetened with sugar) soda pop, regular beverages from powder, and sports drinks.
6	Öhlund et al. 2007 (27)	5-day food records	Frequency (servings/5 days); Quantity (grams/5 days)	Parents (self-administered)	FS: Food groups: Cheese, Black pudding, Candies/sugar, sweet products (includes biscuits, cakes, sweet rolls, ice cream, fruit syrup, other soft drinks, marmalade, jam, chocolate, candies and sugar).
7	Feldens et al. 2010 (28)	Structured interviews at 6 mo and single 24-h dietary recall at 12 mo^1^ (multiple pass method)	Frequency: number of meals and snacks (<7, 7–8, >8) Number of sugary food items: fruit juices/soft drinks in a bottle (yes/no) Percentage: density of sugar (≤ or >50% of simple carbohydrates in 100 g of food)	Mothers (face-to-face interview by Nutrition undergraduate students; at 12 mo 24-h dietary recall recorded by fieldworkers)	FS: 6-mo: Breastfeeding (BF) and bottle-feeding day and night, sugar, honey, sweetened beverages, biscuits, chocolate and salty snacks; 12-mo: frequency of BF, number of meals and snacks, fruit juice, beverage, tea, candies, soft drink, sugar and honey, salty snacks, filled cookies and chocolate.
8	Tanaka et al. 2013 (29)	Self-administered diet history questionnaire	Frequency (never; sometimes or usually)	Mothers (self-administered)	FS: Sweetened liquids other than milk (bottle); Bottle-feeding when falling asleep at night.
9	Chaffee et al. 2015 (7)	Food list to construct three dietary indices: 6 mo sweet dietary index, 6 mo non-sweet dietary index, and 12 mo sweet dietary index	Number of sugary food items (no. of sugar items introduced to the infant before age 6-mo and at 12-mo)	Mothers (interviewed by trained and calibrated fieldworkers)	IS, MS, FS: At 6-mo - cariogenic group: added sugar, candy, chips, chocolate, chocolate milk, coffee (sugar added), cookies, fruit-flavored drinks, gelatine, honey, ice cream, petit Suisse cheese, soft drinks, sweet biscuits, and tea; low sugar group: beans, cow’s milk, commercial soup, enriched cereal, family food, fried foods, fruit, natural fruit juice, processed meat, red meat, organ meat, salty snacks, savory porridge, simple grain or flour, water, and vegetables. At 12-mo - cariogenic group: added sugar in a drink, candy, cake, chips, chocolate, chocolate, chocolate, chocolate milk, coffee (sugar added), cookies, creamed caramel, fruit-flavored drink, gelatine, honey, ice cream, other confections, petit Suisse cheese, soft drinks, and sweet biscuits.
10	Park et al. 2015 (30)	Survey questionnaire	Frequency (times per day or per week)	Mothers (self-administered) (intake in past 7 days)	FS: Sugar-sweetened beverages (SSB): juice drinks, soft drinks, soda, sweet tea, Kool-Aid. Sweet foods included candy, cookies, cake, doughnuts, muffins, and pop-tarts.
11	Wigen and Wang 2015 (31)	Questionnaire	Frequency (Sugary drinks: < 1 time/week, 1–6 times/week or daily. Sugary drinks at night: never, sometimes, each night)	Parents (self-administered)	FS: Sugary drinks: Sugary drinks at night.
12	Peres et al. 2016 (11)	Food list (4 y); Food Frequency Questionnaire (FFQ) (>15 y) (81 foods and 88 drink items)	Frequency @4 year: times/daily, times/weekly, <once a week. at 15y: times/day, times/week, times/month, times/year at 18y: never or <1/month, 1–3/month, 1/week, 2–4/week, 5–6/week, 1/day, 2–4/day, >5/day Number of sugary food items (score 0–11 foods consumed)	at 4 years Mothers (self-administered) at 15- and 18-years old Adolescents (self-administered)	FS: 4y-old-chips, soda, chocolate, candies, bubble gum, and lollypop. 15y and 18y-old- cake, chips/snacks, cookies, ice cream or popsicles, sugar, candies, chocolate in powder or chocolate bars, pudding, non-diet soda, natural fruit juice, and processed fruit juice.
13	Avasare et al. 2017 (32)	Questionnaire^1^	Quantity (ounces/week)	Mothers (self-administered)	MS, FS: Water, infant formula, milk, soda pop, sports drinks, and others. Any sugared beverages: 100% juice, juice drinks, flavored water, sugared beverages made from powder, soda pop, sports drinks, chocolate milk, and other sugared beverages.
14	VanBuren et al. 2017 (33)	Food Frequency Questionnaire (FFQ) (number of items not reported)	Quantity (ounces/day)	Parents (self-administered)	MS, FS: Water, milk, 100 percent juice, and SSB.
15	Feldens et al. 2018 (34)	Two 24-h recall using multiple pass method	Frequency (≤5 times/day, > 5 times/day, mean quintiles of feeding episodes)	Mothers (face to face interviews)	MS, FS: Breastfeeding, Baby-bottle use (including all liquids, such as milk, water, juice, soda or tea) and other foods and drinks.
16	Bell et al. 2019 (35)	single 24-h dietary recall (using a five-step multi-pass method) and single 2-day food record	Energy (calories/day from free sugars)	Mothers (self-administered) (non-consecutive 10-day period including week and weekend days)	IS, MS, FS: Family diet: vegetables, fresh fruit, non-white bread, cheese and non-discretionary red meat and poultry. Cow’s Milk and Discretionary: cow’s milk, fluoridated water, white bread, cheese and discretionary foods, including processed meat, sugary products, SSB and discretionary potato products.
17	Hu, Shijia et al. 2019 (36)	3-day food diary or single 24-h dietary recall (using a 5-stage, multiple-pass interviewing technique)	Quantity (grams/day, milliliters/day) Frequency (times/day)	3-day food diaries: Mothers or caregivers (self-administered) 24-h recall: Mothers or caregivers (interview by trained personnel)	FS: Confectionary food group: chocolates, sweets, ice cream, puddings and jellies. SSB: fruit drinks, carbonated soft drinks, sweetened soya milk, traditional drinks and other sweetened drinks like honey mixed with water.
18	Bernabé et al. 2020 (8)	Survey questionnaire	Frequency (times/day)	Parents (self-administered)	FS: SSBs- soft drinks, fruit drinks, energy and sports drinks, and drinks sweetened after purchase
19	Pitchika et al. 2020 (37)	Food Frequency Questionnaire (FFQ)^1^ (17 major food groups or 41 subgroups)	Quantity (g/day, sugar-sweetened drinks (SSD) portions of 250 mL/day) Energy (kcal/day) (total energy intake)	at 10 years by Parents (self-administered) at 15 years by Children with help of parents (self-administered) (intake in past 12 months)	FS: Sugar-Sweetened Drinks (SSD)- Colas, lemonades, iced tea, sport/energy drinks, fruit squashes and nectars.
20	Carvalho Silva et al. 2021 (38)	Food Frequency Questionnaire (FFQ)^1^ (35 food items)	Frequency (never to ≥4 times per day)	Parents or caregivers (trained interviewers) (intake in past 6 months)	FS: Cariogenic foods-ice cream, breakfast cereals, crackers, cookies, sweet pastry, chocolate, sugar, and candies. Cariogenic drinks- sweetened carbonated drinks and other SSD.
21	Feldens et al. 2021 (39)	Food list	Number of sugary food items (number of sugar items introduced to the infant before age 6-mo) Quantity (kg/person household sugar purchase monthly at 3y)	Mothers (interviewer administered)	FS: 6-mo: added sugar, candy, chips, chocolate, chocolate milk, coffee with sugar added, cookies, fruit-flavored drinks, gelatine, honey, ice cream, petit Suisse cheese, soft drinks, sweet biscuits, and tea with sugar added.
22	Manohar et al. 2021 (40)	Short Food Frequency Questionnaire (FFQ)^2^ (32 food and drink items)	Frequency (times/week)	Mothers (telephone interviews) (intake in past 7 days)	FS: Foods high in saturated fats- biscuits, cakes, puddings, potato chips and savory snacks. Foods and drinks with added sugars- confectionary, SSB, flavored mineral water, sports drinks, sweetened yogurt, ice cream, and syrups or spreads.
23	Marshall et al. 2021 (13)	Semi-quantitative Food Frequency Questionnaire (FFQ)^1^ (4 beverage categories)	Quantity (ounces per day)	Parents (self-administered) (intake in previous week)	MS, FS: Cows’ milk (including infant formulas and flavored milk), juice (including liquid juice drinks before age 9 years), SSB, liquid juice drinks after age 9 years, and water/sugar-free beverages (water/ SFB).
24	Moreira et al. 2021 (41)	Food Frequency Questionnaire (FFQ)^1^ (106 food items)	Percentage (daily added sugar intake <10%, 10–20%, ≥ 20%)	Adolescents (interview) (intake in last 12 months)	MS, FS: Soft drinks, fruit-flavored juice, chocolate drinks, energy drinks, dairy products, bread, cookies, breakfast cereals, desserts, chocolate, mayonnaise, salty snacks, and cold cuts.
25	Boustedt et al. 2022 (42)	Structured questionnaire	Frequency (times/day)	Parents or caretakers (self-administered)	FS: Drinks with meals, frequency of nightly and in-between meals, intake of ice cream, sweets/candy and fast food.
26	Echeverria et al. 2022 (43)	Food list	Number of sugary food items (yes/no)	Caregiver (trained interviewers) (intake in last 24-h)	FS: 3-mo: sugar or honey; sugar, honey, chocolate milk 12-mo: drink sweetened with sugar 24-mo: juice in a box or bottled juice or powdered juice or bottled coconut, water or coconut water in a box, soft drink, sweet cookie, candies, lollipops, chewing gums, chocolates, or jelly 24 to 48-mo: sugar or honey in drinks such as milk, tea, or juice, chocolate milk, sugar or honey in fruits.
27	Ha et al. 2022 (44)	Food list	Frequency (every day, sometimes, never) Number of sugary food items (summed to composite score for proxy of free sugars intake)	Mothers (self-administered) (intake in previous week)	FS: Soft/soda drinks containing sugars, flavored milk, tea/coffee with added free sugars
28	Wu, Tong et al. 2022 (45)	Structured questionnaire	Frequency (never/rarely; sometimes; ≥1 per day)	Mothers or caregivers (self-administered)	FS: Fruit juice, snacks, gum, lollipop candy, dried fruit, soft drink.
29	da Silva et al. 2023 (46)	Food Frequency Questionnaire (FFQ)^1^ (24 ultra-processed food items)	Frequency(times/day) Quantity (grams/day) Energy (total calorie intake/day)	Guardians (interview) (intake in previous 12 months)	MS, FS: Biscuits, savory snacks and sugar-sweetened cereals: sweet biscuits/filled biscuits/cookies, salted biscuits, crisps/savory snacks, granola, breakfast cereals and cereal bars. Ultra-processed meats and fats: sausage or frankfurter, hot dog sausage, mortadella, ham, salami, processed hamburgers or nuggets, butter/margarine, mayonnaise and cream cheese. Sweets: ice cream or popsicles, candy or lollipop and chocolate bar or bonbon. Fast food and instant noodles: grilled sandwich/cheeseburger/hot dog, Nissin Miojo®/cup noodles and pizza. Soft drinks and artificially flavored juices: regular soft drinks, diet/light soft drinks and artificial juice (liquid or powder mix). Sweetened milk and powdered chocolate: dairy products and powdered chocolate or Nescau^®^.
30	Echeverria et al. 2023 (47)	Food list^1^	Number of sugary food items (yes/no)	Mothers or guardians (interviewer administered) (intake in last 24-h)	FS: 3-mo: sugar or honey; sugar, honey, chocolate milk 12-mo: drink sweetened with sugar 24-mo: juice in a box or bottled juice or powdered juice or bottled coconut, water or coconut water in a box, soft drink, sweet cookie, candies, lollipops, chewing gums, chocolates, or jelly 24 to 48-mo: sugar or honey in drinks such as milk, tea, or juice, chocolate milk, sugar or honey in fruits.
31	Ha et al. 2023 (48)	single 24-h recall (using the multipass method) and 2-day diet diary (1 y) and Food Frequency Questionnaire (FFQ) ^1^ (2y, 5y) (age-specific 98- and 99-items)	Quantity (g) (free sugar intake in g was calculated)	Mothers (self-administered for 2-day diet diary/FFQ and telephone interviews for 24-h recall) (intake in two weekdays and one weekend day)	IS, MS, FS: Milk and milk alternatives, condensed and evaporated milk, cheese, yogurt, cream and custard, frozen dessert, fruits, dried fruits, tinned fruit, pureed fruits and vegetables, nuts, nut pastes and other spreads, breakfast cereal, biscuits, bars and bar like snack food, cakes and puddings, sweet bread and pastry, savory snack food, drinks, drink powder, sauces and condiments, sugar and sugar substitutes, jelly, chocolates and lollies (68)
32	Alkadi et al. 2024 (49)	Questionnaire^3^	Number of sugary food items (Sweet index: 0–8, non-sweet index: 0–7)	Mothers (self-administered)	IS, MS, FS: High cariogenic: food items including chips, crackers, cookies, candy, soda or diet soda, dried fruit, ice cream, and fruit drinks with sugar. Low cariogenic: food items including yogurt, dry cereal, fresh fruit, water, 100% juice, fruit drinks without sugar, and milk.
33	Mathias et al. 2024 (50)	Food list	Number of sugary food items (Ultra-Processed Foods consumption: yes/no Score: low/medium <7, high ≥7)	Parents or caregivers (interviewer administered) (habitual consumption)	FS: Ultra-Processed Foods (UPF): Carbonated soft drinks; chocolate powder in milk; nuggets, hamburgers, or sausages; packaged salty snacks; candies, lollipops, chewing gum, chocolate, or jelly; salty crackers; sweet biscuits; juice in a box or prepared from a powdered mix; and yogurt.
34	Kerguen et al. 2025 (51)	Food Frequency Questionnaire (FFQ)^1^ (38 food items)	Frequency (never, once, several times, often, every day or almost every day)	Parents (self-administered)	FS: Fruit Juices and SSB (including syrup, baby tea, soda)

1 Validated/piloted study reported in the article.

2 Previous studies (69–74).

3 Previous studies (7, 11).

## 4 Discussion

This comprehensive scoping review identified various dietary assessment methods for measuring sugar intake in relation to oral health outcomes in OHBCS. FFQs, food lists, food diaries, 24-h recalls, and structured interviews were used to assess the sugar intake in OHBCS. FFQ was the most common assessment method reported in OHBCS. The review included OHBCS from a wide range of countries, with Brazil and the United States making the largest contributions. This is likely due to the presence of long-standing cohorts, such as the Pelotas Birth Cohort Study and the Iowa Fluoride Study. The sample sizes in the selected articles varied widely, which may have affected the choice of dietary assessment. For example, measuring sugar intake in 86 participants (27) is considerably less burdensome than 10,921 participants (51). Since dietary assessment methods are designed for specific populations or countries, they should be adapted, evaluated, and re-validated when applied to different settings.

The Food Frequency Questionnaire emerged as the most commonly used dietary assessment method, likely due to its practicality and ability to capture dietary patterns over extended periods (52). Nevertheless, these questionnaires are prone to recall and social desirability biases, may impose a burden on participants, and often rely on individuals’ literacy and physical capabilities (53). Despite these limitations, FFQs effectively assess overall dietary intake, which is essential for examining long-term associations between sugar intake and non-communicable diseases, including dental caries and other oral health conditions. In this review, studies administered dietary instruments ranging from four beverage categories (13) to 106 food items (41), with only a few capturing the daily frequency of sugar intake (11, 38, 40, 46, 51), a crucial factor for oral health. No study has reported using digital devices to collect dietary information on participants’ diets.

Researchers have widely used non-specific questionnaires; however, their limited focus on sugars prevents reliable estimation of the quantity or frequency of sugar consumption. Moreover, questionnaires with restricted food items may lead to under- or misreporting of sugar consumption (54, 55). To address these challenges, alternative methods like food diaries and records have been explored for more reliable dietary assessment.

Food diaries and records as prospective dietary assessment methods are more accurate due to lower recall bias and real-time data collection compared to retrospective methods like FFQs and dietary recalls. Still, food diaries and records are time-consuming and challenging for both respondents and researchers and may influence habitual intake. Several studies estimated sugar intake using food records ranging from 2 to 5 days.

The 24-h recall, an in-depth interview conducted by a trained dietary interviewer, is pivotal for capturing accurate and complete dietary data (52). All studies used the multiple-pass method to collect dietary data, with recall periods ranging from single-day to 2-day assessments. However, the accuracy of this method heavily depends on the participant’s memory and the interviewer’s skill in probing for precise portion sizes. Although a 24-h recall over two consecutive days reduces within-person variation, some nutrients and food groups may require up to 7 days of data collection for accurate assessment (52).

The emphasis on measuring sugar intake frequency rather than its quantity, caloric contribution, or percentage of total consumption may limit the ability to fully capture the multifaceted relationship between sugars and oral health outcomes. However, the importance of both the frequency and quantity of sugars consumed is well-known (6, 56). The variability in measurement approaches emphasizes a lack of consensus and highlights the challenges of assessing sugars’ impact on oral health in the existing evidence. The lack of standardization in dietary assessment methods found in this review may be influenced by factors such as population literacy levels, study objectives, and available resources. For instance, some studies focused on dietary behaviors, patterns, or trajectories (24, 25, 38–40, 43, 48) and their long-term implications for chronic diseases over time. Conversely, others examined early-life feeding practices (7, 28, 29) or initial sugar exposure (8, 42, 47, 51) and their direct association with dental caries. Considering both the amount of sugar intake (g/day or percentage contribution to energy intake) as well as its frequency may provide valuable insights into the ongoing debate regarding their relative importance in oral health research. Standardizing these measurement methods would enhance comparability across studies and strengthen the evidence base for public health recommendations.

A key limitation of the reviewed studies is the lack of reporting validation or piloting of sugar intake assessment methods (7, 8, 11, 12, 26, 27, 30, 31, 33–36, 39, 42–45, 50), which raises concerns about data reliability. Validating dietary assessments using biomarkers or reference methods (57), such as comparing reported energy intake to basal metabolic rate (BMR) and physical activity level (PAL) (58), is cardinal, yet challenging, to assess in young children. On a related note, self-reported methods are prone to measurement or information bias (43, 59), but digital tools, such as Intake24 (60) and ASA24 (Automated Self-Administered 24-Hour Dietary Assessment Tool) (61), offer more accurate real-time data collection.

Studies assessed various sugars, with a predominant focus on free sugar intake and oral health. Standardizing sugar intake measures in oral health birth cohort studies (OHBCS) is crucial, and reporting intake as a percentage of total energy enables comparisons with WHO guidelines and other authoritative bodies is recommended. Transparency in funding and conflicts of interest is essential (62), yet several studies omitted disclosures (12, 23–27, 29, 32), while some had sugar industry funding (21, 22) or professional conflicts of interest (36).

Pooling sugar intake data remains challenging due to methodological inconsistencies (63), complicating dietary guidelines development (64). Standardized approaches must account for factors such as age, socioeconomic status, and geographic location (17, 47, 65). To accurately assess sugars’ impact on oral health, key confounders—including socioeconomic status, breastfeeding, fluoride exposure, and oral hygiene behaviors (56, 66)—must be considered (covariates adjusted for statistical analysis are presented in Supplementary Table 4).

Limitations of this review include the exclusion of studies where dietary sugars were analyzed as a covariate, as this information was often unavailable in titles and abstracts, necessitating a full-text review of nearly all OHBCS studies. Additionally, study quality was not assessed, in line with the scoping review methodology. On the other hand, this review employed a comprehensive search strategy across major databases, hand-searched reference lists, and followed PRISMA-ScR guidelines. The adopted approach provided a broad, global perspective on assessment methods used to measure sugar intake in OHBCS by not restricting the search by language or publication date. Although the grey literature was not included, this gap was mitigated by updating the search and manually reviewing the reference lists of the included studies. While this review provides an overview of assessment methods used to measure sugar intake, it does not address the associations between sugar consumption and oral health outcomes, which remain beyond its scope.

## 5 Conclusion

Various dietary assessment methods, such as FFQs, food lists, food diaries, 24-h recall, and structured interviews, have been used to assess sugar intake in OHBCS. FFQ was the most common assessment method reported in FFQ was the most common assessment method reported in OHBCS to measure sugar intake. We recommend that future studies should use validated dietary assessment methods, such as FFQs, to ensure accurate estimation of long-term sugar intake. A standardized approach to harmonize dietary sugars data will guide early interventions, inform dietary recommendations, facilitate comparisons, and improve data pooling across birth cohort studies.
