# Supplementary material for: Measuring sugar intake in oral health birth cohort studies: a scoping review
Source: Front Nutr. 2026 Jan 7;12:1667487. doi: 10.3389/fnut.2025.1667487 (PMC12821232; doi:10.3389/fnut.2025.1667487)
Supplement: Supplementary file 1 [file Table_1.docx]

**Supplementary Table 1.** Search strategy

| **Database** | **Search terms** | **Hits** | **Date of search** |
| --- | --- | --- | --- |
| **Dentistry & Oral Sciences Source** | ( birth* OR pregnan* OR prenatal OR antenatal OR infan* OR (infant OR newborn ) AND ( 'longitudinal analys*' OR 'longitudinal design*' OR 'longitudinal evaluation*' OR 'longitudinal research' OR 'longitudinal studies' OR 'longitudinal study' OR 'longitudinal survey*' OR 'follow up evaluation*' OR 'followup evaluation*' OR 'followup stud*' OR 'follow up stud*' OR 'followup survey*' OR 'follow up survey*' OR 'prospective analys*' OR 'prospective evaluation*' OR 'prospective studies' OR 'prospective study' OR 'prospective survey*' OR cohort*) ) AND ( 'oral health' OR 'tooth loss*' OR 'decayed missing and filled teeth' OR dmf* OR edent* OR 'dental status*' OR periodontitis OR periodont* OR caries OR tooth* OR 'dental pain' OR dental* OR dentition* OR 'oral cancer' OR 'mouth neoplasms' OR malocclusion OR teeth OR xerostomia) ) AND ( diet* OR intake* OR consumption* OR sugar* OR saccharide* OR sugar sweetened beverage* OR juice* OR honey OR sugary snack* OR sweet* OR cand* OR soft drink* OR fizzy drink* OR carbonated drink* OR carbonated beverage* OR confectioner* OR cariogenic OR syrup*) ) | 252 | **17 June 2025** |
| **Embase** | (diet* OR dietary OR intake* OR consumption* OR sugar* OR saccharide OR saccharides OR monosaccharide OR monosaccharides OR disaccharide OR disaccharides OR sucrose OR maltose OR lactose OR trehalose OR glucose OR fructose OR galactose OR sugar sweetened beverage* OR juice* OR honey OR sugary snack* OR sweet* OR cand* OR soft drink* OR fizzy drink* OR carbonated drink* OR carbonated beverage* OR confectioner* OR cariogenic OR syrup*) AND (birth*:ti,ab OR pregnan*:ti,ab OR prenatal:ti,ab OR antenatal:ti,ab OR infan*:ti,ab OR (infant,:ti,ab AND newborn:ti,ab)) AND ('longitudinal analys*':ti,ab OR 'longitudinal design*':ti,ab OR 'longitudinal evaluation*':ti,ab OR 'longitudinal research':ti,ab OR 'longitudinal studies':ti,ab OR 'longitudinal study':ti,ab OR 'longitudinal survey*':ti,ab OR 'follow up evaluation*':ti,ab OR 'followup evaluation*':ti,ab OR 'followup stud*':ti,ab OR 'follow up stud*':ti,ab OR 'followup survey*':ti,ab OR 'follow up survey*':ti,ab OR 'prospective analys*':ti,ab OR 'prospective evaluation*':ti,ab OR 'prospective studies':ti,ab OR 'prospective study':ti,ab OR 'prospective survey*':ti,ab OR cohort*:ti,ab) AND ('oral health':ti,ab OR 'tooth loss*':ti,ab OR 'decayed missing and filled teeth':ti,ab OR dmf*:ti,ab OR edent*:ti,ab OR 'dental status*':ti,ab OR periodontitis:ti,ab OR periodont*:ti,ab OR caries:ti,ab OR tooth*:ti,ab OR 'dental pain':ti,ab OR dental*:ti,ab OR dentition*:ti,ab OR 'oral cancer':ti,ab OR 'mouth neoplasms':ti,ab OR malocclusion:ti,ab OR teeth:ti,ab OR xerostomia:ti,ab) | 52 |  |
| **PubMed** | (diet* OR dietary OR intake* OR consumption* OR sugar* OR saccharide OR saccharides OR monosaccharide OR monosaccharides OR disaccharide OR disaccharides OR sucrose OR maltose OR lactose OR trehalose OR glucose OR fructose OR galactose OR sugar sweetened beverage* OR juice* OR honey OR sugary snack* OR sweet* OR cand* OR soft drink* OR fizzy drink* OR carbonated drink* OR carbonated beverage* OR confectioner* OR cariogenic OR syrup*) AND  (Birth*[tiab] OR pregnan*[tiab] OR prenatal [tiab] OR antenatal [tiab] OR infan* [tiab] OR Infant, Newborn [mh]) AND (longitudinal studies[mh] OR longitudinal analys*[tiab] OR longitudinal design*[tiab] OR longitudinal evaluation*[tiab] OR longitudinal research[tiab] OR longitudinal studies[tw] OR longitudinal study[tw] OR longitudinal survey*[tiab] OR follow up evaluation*[tiab] OR followup evaluation*[tiab] OR followup stud*[tiab] OR follow up stud*[tiab] OR followup survey*[tiab] OR follow up survey* [tiab] OR prospective analys*[tiab] OR prospective evaluation*[tiab] OR prospective studies[tw] OR prospective study[tw] OR prospective survey*[tiab] OR cohort*[tiab]) AND (Oral Health[TIAB] OR Tooth Loss*[TIAB] OR Decayed Missing and Filled Teeth[TIAB] OR DMF*[TIAB] OR Edent*[TIAB] OR Dental Status*[TIAB] OR Periodontitis [MH] OR Periodont* [TIAB] OR Caries [TIAB] OR Tooth* [TIAB] OR Dental pain [TIAB] OR dental* [TIAB] OR Dentition* OR oral cancer [TIAB] OR Mouth Neoplasms [MH] OR Malocclusion [TIAB] OR Teeth [TIAB] OR Xerostomia [TIAB]) | 415 |  |
| **Scopus** | diet* OR intake* OR consumption* OR sugar* OR saccharide* OR monosaccharide*or AND disaccharide* OR sucrose OR maltose OR lactose OR trehalose OR glucose OR fructose OR galactose OR beverage* OR juice* OR honey OR snack* OR sweet* OR cand* OR drink* OR confectioner*or AND cariogenic AND food* OR syrup* AND birth* OR pregnan* OR prenatal OR antenatal OR infan* OR infant OR newborn AND longitudinal AND studies OR longitudinal AND analys* OR longitudinal AND design* OR longitudinal AND evaluation* OR longitudinal AND research OR longitudinal AND studies OR longitudinal AND study OR longitudinal AND survey* OR follow AND up AND evaluation* OR follow AND up AND evaluation* OR follow AND up AND stud* OR follow AND up AND stud* OR follow AND up AND survey* OR follow AND up AND survey* OR prospective AND analys* OR prospective AND evaluation* OR prospective AND studies OR prospective AND study OR prospective AND survey* OR cohort* AND oral AND health OR tooth AND loss* OR decayed AND missing AND filled AND teeth OR dmf* OR edent* OR dental AND status* OR periodontitis/ OR periodont* OR caries OR tooth* OR dental AND pain OR dental* OR dentition* OR oral AND cancer OR mouth AND neoplasms/ OR malocclusion OR teeth OR xerostomia | 8 |  |
| **Web of Science** | TS=(birth* OR pregnan* OR prenatal OR antenatal OR infan* OR (infant AND newborn)) AND TS= ('longitudinal analys*' OR 'longitudinal design*' OR 'longitudinal evaluation*' OR 'longitudinal research' OR 'longitudinal studies' OR 'longitudinal study' OR 'longitudinal survey*' OR 'follow up evaluation*' OR 'followup evaluation*' OR 'followup stud*' OR 'follow up stud*' OR 'followup survey*' OR 'follow up survey*' OR 'prospective analys*' OR 'prospective evaluation*' OR 'prospective studies' OR 'prospective study' OR 'prospective survey*' OR cohort*) AND TS= ('oral health' OR 'tooth loss*' OR 'decayed missing and filled teeth' OR dmf* OR edent* OR 'dental status*' OR periodontitis OR periodont* OR caries OR tooth* OR 'dental pain' OR dental* OR dentition* OR 'oral cancer' OR 'mouth neoplasms' OR malocclusion OR teeth OR xerostomia) AND TS= (diet* OR intake* OR consumption* OR sugar* OR saccharide* OR sugar sweetened beverage* OR juice* OR honey OR sugary snack* OR sweet* OR cand* OR soft drink* OR fizzy drink* OR carbonated drink* OR carbonated beverage* OR confectioner* OR cariogenic OR syrup*) | 924 |  |
