# Supplementary material for: Measuring sugar intake in oral health birth cohort studies: a scoping review
Source: Front Nutr. 2026 Jan 7;12:1667487. doi: 10.3389/fnut.2025.1667487 (PMC12821232; doi:10.3389/fnut.2025.1667487)
Supplement: Supplementary file 2 [file Table_2.docx]

| **N°** | **Author/Year** | **Inclusion/Exclusion** | **Reasons** |
| --- | --- | --- | --- |
| 1 | *Aaltonen et al. 1994 (1)* | Excluded | Sugar is not main exposure |
| 2 | *Marshall et al. 2004 (2)* | Excluded | Sugar as confounder |
| 3 | *Maupome et al. 2010 (3)* | Excluded | Sugar data not collected |
| 4 | *Arora et al. 2011 (4)* | Excluded | Study Proposal |
| 5 | *Warren et al. 2016 (5)* | Excluded | Sugar is not main exposure |
| 6 | *Peres et al. 2017 (6)* | Excluded | Sugar as confounder |
| 7 | *Devenish et al. 2020 (7)* | Excluded | Sugar as confounder |
| 8 | *Carpenter et al. 2021 (8)* | Excluded | No oral health outcome |
| 9 | *Carvalho et al. 2021 (9)* | Excluded | Sugar is not main exposure |
| 10 | *George et al. 2021 (10)* | Excluded | No oral health outcome |
| 11 | *Manohar et al. 2021 (11)* | Excluded | No oral health outcome |
| 12 | *Abanto et al. 2023 (12)* | Excluded | Sugar as mediator |
| 13 | *Chisini et al. 2023 (13)* | Excluded | Sugar as mediator |

Supplementary material:

Supplementary Table 2. Excluded reports and reasons for exclusion.

**References**

1. Aaltonen AS, Tenovuo J. Association between mother-infant salivary contacts and caries resistance in children: a cohort study. Pediatr Dent. 1994;16(2):110-6.

2. Marshall TA, Levy SM, Warren JJ, Broffitt B, Eichenberger-Gilmore JM, Stumbo PJ. Associations between Intakes of fluoride from beverages during infancy and dental fluorosis of primary teeth. J Am Coll Nutr. 2004;23(2):108-16.

3. Maupomé G, Karanja N, Ritenbaugh C, Lutz T, Aickin M, Becker T. Dental caries in American Indian toddlers after a community-based beverage intervention. Ethn Dis. 2010;20(4):444-50.

4. Arora A, Scott JA, Bhole S, Do L, Schwarz E, Blinkhorn AS. Early childhood feeding practices and dental caries in preschool children: a multi-centre birth cohort study. BMC Public Health. 2011;11:28.

5. Warren JJ, Blanchette D, Dawson DV, Marshall TA, Phipps KR, Starr D, Drake DR. Factors associated with dental caries in a group of American Indian children at age 36 months. Community Dent Oral Epidemiol. 2016;44(2):154-61.

6. Peres KG, Nascimento GG, Peres MA, Mittinty MN, Demarco FF, Santos IS, et al. Impact of Prolonged Breastfeeding on Dental Caries: A Population-Based Birth Cohort Study. Pediatrics. 2017;140(1).

7. Devenish G, Mukhtar A, Begley A, Spencer AJ, Thomson WM, Ha D, et al. Early childhood feeding practices and dental caries among Australian preschoolers. Am J Clin Nutr. 2020;111(4):821-8.

8. Carpenter L, Gibbs L, Magarey A, Dashper S, Gussy M, Calache H. Nutrition and oral health in early childhood: associations with formal and informal childcare. Public Health Nutr. 2021;24(6):1438-48.

9. Carvalho Silva C, Gavinha S, Manso MC, Rodrigues R, Martins S, Guimarães JT, et al. Serum Levels of Vitamin D and Dental Caries in 7-Year-Old Children in Porto Metropolitan Area. Nutrients. 2021;13(1).

10. George A, Villarosa AR, Ingram S, Fatema K, Elliott K, Grace R, et al. Oral health status, behaviours, food and beverage consumption of aboriginal children in Australia. Health Promot J Austr. 2021;32(2):208-15.

11. Manohar N, Hayen A, Do L, Scott J, Bhole S, Arora A. Early life and socio-economic determinants of dietary trajectories in infancy and early childhood – results from the HSHK birth cohort study. Nutrition Journal. 2021;20(1):76.

12. Abanto J, Maruyama JM, Pinheiro E, Matijasevich A, Antunes JLF, Bönecker M, Cardoso MA. Prolonged breastfeeding, sugar consumption and dental caries at 2 years of age: A birth cohort study. Community Dent Oral Epidemiol. 2023;51(3):575-82.

13. Chisini LA, Costa FDS, Horta BL, Tovo-Rodrigues L, Demarco FF, Correa MB. Sweet Taste Receptor Gene and Caries Trajectory in the Life Course. J Dent Res. 2023;102(4):422-30.
