# Supplementary material for: Measuring sugar intake in oral health birth cohort studies: a scoping review
Source: Front Nutr. 2026 Jan 7;12:1667487. doi: 10.3389/fnut.2025.1667487 (PMC12821232; doi:10.3389/fnut.2025.1667487)
Supplement: Supplementary file 3 [file Table_3.docx]

| **Supplementary Table 3. Statistical tests used, main findings and source of funding of selected articles.** | | | | | |
| --- | --- | --- | --- | --- | --- |
| **N°** | **Author/Year** | **Type of statistical analysis** | **Main Findings** | **Funding** |  |
| 1 | *MacKeown et al. 2000 (23)* | dmfs: mean/median analysis between groups (Kruskal-Wallis non-parametric test); Trend of caries prevalence (Mantel-Haenszel chi-square test); General linear model and Pearson correlations. | At age 5, the cross-sectional analysis showed a notable increase in caries prevalence and dmfs scores associated with a higher intake of energy, carbohydrates, and added sugars. However, no significant changes were observed in the longitudinal sample. | Not specified |  |
| 2 | *Habibian et al. 2001 (24)* | Student t-test and Pearson product tests | There was no significant association between plaque at 12 or 18 months and the average daily frequency of consuming foods and drinks containing non-milk extrinsic sugars (NMES) at any age. A positive association between the average number of daily eating or drinking episodes and plaque was unaffected by tooth brushing. | The Sugar Bureau and Cow & Gate Ltd. |  |
| 3 | *Habibian et al. 2002 (25)* | Mann–Whitney and Spearman rank correlation tests; χ2 or Fisher’s exact test; Multivariable logistic regression | The presence of *S. mutans* showed a significant correlation with the total number of daily eating and drinking occasions and was nearly significantly associated with the average daily intake frequency of foods and drinks containing NMES. | The Sugar Bureau and Cow & Gate Ltd. |  |
| 4 | *Warren et al. 2002 (26)* | Bivariate chi-square analyses, t-tests, bivariate Pearson correlation coefficients | No statistically significant associations were found between tooth wear and the consumption of soft drinks or juices at any time point. | Not specified |  |
| 5 | *Marshall et al. 2003 (12)* | Wilcoxon rank, Simple Logistic Regression, Multivariable Logistic and Censored Regression Analyses | A high consumption of soda pop and powdered beverages is associated with a greater caries experience, while milk showed no significant association with dental caries. | National Dairy Council, the National Institute for Dental and Craniofacial Research and the General Clinical Research Centres. |  |
| 6 | *Öhlund et al. 2007 (27)* | Spearman correlation; Stepwise logistic regression | Caries experience showed no correlation with the frequency or total intake of biscuits, cakes, sweet rolls, ice cream, fruit syrup, soft drinks, marmalade, jam, chocolate, candies, or sugar. | Swedish Medical Research Council; Swedish Research Council for Environment, Agricultural Sciences and Spatial Planning (FORMAS); Swedish Nutrition Foundation; Sven Jerring Foundation; Samariter Foundation; Oskar Foundation. |  |
| 7 | *Feldens et al. 2010 (28)* | Multivariable Poisson regression | The prevalence of S-ECC was 1.4 times greater among those with the highest consumption of foods and beverages high in added sugars. | Not specified |  |
| 8 | *Tanaka et al. 2013 (29)* | Multivariable logistic regression | Using bottle feeding for sweetened liquids, other than milk, raises the likelihood for caries development. | Kakenhi and Health and Labour Sciences research grants; Research on Allergic Disease and Immunology/Ministry of Health, Labour, and Welfare, Tokyo, Japan |  |
| 9 | *Chaffee et al. 2015 (7)* | Log-linear regression models (S-ECC); negative binomial regression models (dmft) | S-ECC was 1.5 and dmft was 1.8 times higher at the earliest level of sugar introduction. | NIH National Institute for Dental and Craniofacial Research (F30DE022208); NIH National Centre for Advancing Translational Sciences (KL2TR000143); Rio Grande do Sul Research Support Foundation (FAPERGS) |  |
| 10 | *Park et al. 2015 (30)* | Multivariable logistic regression | Amongst 6-year-old children, frequent SSB intake at 10–12 months of age significantly increased the likelihood of having dental caries. | Not specified |  |
| 11 | *Wigen and Wang 2015 (31)* | Multivariable logistic regression models | Consuming sugary drinks at least once a week at 1.5 years old increases the likelihood of developing caries lesions by the age of 5. | Norwegian Ministry of Health and the Ministry of Education and Research, the Norwegian Research Council/FUGE |  |
| 12 | *Peres et al. 2016 (11)* | Generalized linear mixed model | The higher the sugar consumption along the life course, the higher the dental caries increment. | Welcome Trust/Major Awards for Latin America on Health Consequences of Population Change; European Union; National Support Program for the Centres of Excellence; Brazilian National Research Council; Brazilian National Council for Scientific and Technological Development |  |
| 13 | *Avasare et al. 2017 (32)* | Multivariable logistic regression | Children who drank sports drinks at 9-mo were about 4 times more likely to develop *S. mutans* colonization at the age of 18-mo. | Not specified |  |
| 14 | *VanBuren et al. 2017 (33)* | Multinomial logistic regression | A reduction in the average total ounces of juice consumed per day was observed from the low DFS cluster to the medium DFS cluster and then to the high DFS cluster. | National Institutes of Health, the Roy J. Carver Charitable Trust, and Delta Dental of Iowa Foundation. |  |
| 15 | *Feldens et al. 2018 (34)* | Multivariable Poisson regression | More frequent feeding, including increased daily bottle use, breastfeeding, and consumption of other foods and/or drinks at 12 months of age, was related to a higher prevalence of dental caries in early childhood. | NIH National Institute for Dental and Craniofacial Research (F30DE022208), the NIH National Centre for Advancing Translational Sciences (KL2TR000143), the Rio Grande do Sul Research Support Foundation (FAPERGS), and the Coordination for the Improvement of Higher Education Personnel (CAPES) |  |
| 16 | *Bell et al. 2019 (35)* | Multivariable logistic regression | Discretionary sugars as dietary pattern was not associated with dental caries. | National Health and Medical Research Council (1046219, 1144595, 1101675) |  |
| 17 | *Hu, Shijia et al. 2019 (36)* | Univariate logistic regression and generalized estimating equation (GEE) | No significant association with frequency/amount of SSB and ECC was found | National Institute for Health Research through the NIHR Southampton Biomedical Research Centre. National University Health System under its NUHS Bridging Singapore’s National Medical Research Council |  |
| 18 | *Bernabé et al. 2020 (8)* | Linear Mixed-Effects Models | The initial SSB intake and the deviation from the initial SSB intake were positively associated with steeper caries trajectories. | Chief Scientist Office of the  Scottish Office Department of Health (grant K/OPR/2/2/DTSO) |  |
| 19 | *Pitchika et al. 2020 (37)* | Simple and multivariable logistic regression models | The consumption of sugar-sweetened drinks (SSDs) was associated with higher caries experience at 10 and 15 years of age. | Federal Ministry for Education, Science, Research and Technology and from Helmholtz Zentrum Munich (formerly GSF). German Research Foundation (Deutsche Forschungsgemeinschaft) |  |
| 20 | *Carvalho Silva et al. 2021 (38)* | Bivariate analysis, Multivariable logistic regression | Cariogenic foods and beverages (added-sugar beverages) were not associated to the development of new caries lesions in the three years of follow-up | Regional Department of Ministry of Health Portuguese Foundation for Science and Technology (FCT) and from the Calouste Gulbenkian Foundation. |  |
| 21 | *Feldens et al. 2021 (39)* | Multivariable logistic regression | Children who consumed sugar before 6 months of age were 24% more likely to have caries at 6 years old. | NIH National Institute for Dental and Craniofacial Research (F30DE022208); Rio Grande do Sul Research Support Foundation (FAPERGS); Coordination for the Improvement of Higher Education Personnel (CAPES) |  |
| 22 | *Manohar et al. 2021 (40)* | Group-Based Trajectory Modelling (GBTM) analysis. Multi-level multivariable regression modelling | No association was found between frequent consumption of sugary foods and ECC. | NSW Health, Australian Dental Research Foundation, Western Sydney University, and Oral Health Foundation. |  |
| 23 | *Marshall et al. 2021 (13)* | Univariable generalized linear models Multivariable models | Juice and sugar free beverage (water) intakes were associated with lower caries at age 17 when frequent toothbrushing was present. High consumption of SSB was associated with an increased risk of caries. | National Institutes of Health, The Roy J. Carver Charitable Trust, and Delta Dental of Iowa Foundation. |  |
| 24 | *Moreira et al. 2021 (41)* | Multinomial logistic regression models | Higher added sugar intake was associated with a greater severity of periodontal disease in adolescents. | Maranhão State Foundation for Research and Scientific and Technological Development; National Council for Scientific and Technological Development; Coordination for the Improvement of Higher Education Personnel |  |
| 25 | *Boustedt et al. 2022 (42)* | Chi-square tests and relative risk (RR) with 95% confidence intervals. | Free sugars intake during early life doubled risk for ECC development. | Region Halland, Sweden and the author’s institutions. |  |
| 26 | *Echeverria et al. 2022 (43)* | Generalized estimating equations | Highest prevalence of dental caries was found among children with increasing and always high sugar consumption. | Wellcome Trust; National Council for Scientific and Technological Development (CNPq); Foundation for Research Support of the State of Rio Grande do Sul (FAPERGS); Brazilian Coordination for the Improvement of Higher Education Personnel (CAPES PRINT) |  |
| 27 | *Ha et al. 2022 (44)* | Multivariable regression analysis | Children of mothers in the ‘High early’ and ‘Moderate but increasing’ groups had higher likelihood for increased dmfs scores compared to children of mothers in the ‘Stable low’ group. | Australian National Health and Medical Research Council (APP1046219 2013-17, APP144595 2018-22) |  |
| 28 | *Wu, Tong Tong et al. 2022 (45)* | Multivariable logistic regression model | Caries-free children were found to consume less frequently soft drinks, snacks, lollipop, and other candies. | National Institute of Dental and Craniofacial Research |  |
| 29 | *da Silva et al. 2023 (46)* | Poisson regression models | Daily intake of sweets was associated with increased dental caries burden. Also, sweetened milk and powdered chocolate was associated with dental caries at 12-13 y. | Brazilian Coordination for the Improvement of Higher Education Personnel. Brazilian Public Health Association (ABRASCO). Wellcome Trust. World Health Organization, National Support Program for Centers of Excellence (PRONEX), Brazilian National Research Council (CNPq), Brazilian Ministry of Health and Children's Pastorate. National Research Council (CNPq), protocol number 421044/2018-7. |  |
| 30 | *Echeverria et al. 2023 (47)* | Multivariable Poisson regression | The introduction of added sugars before 12 months of age, increases the chances of developing caries. | Wellcome Trust; National Council for Scientific and Technological Development (CNPq); Foundation for Research Support of the State of Rio Grande do Sul (FAPERGS); Pastoral da Criança; Bernard van Leer Foundation); Brazilian Coordination for the Improvement of Higher Education Personnel (Finance Code 001). |  |
| 31 | *Ha et al. 2023 (48)* | Generalized linear regression models, generalized linear modelling for the negative binomial distribution | Children in the “high and increasing” sugar intake trajectory had higher prevalence and experience of dental caries compared to children in the “low and increasing” sugar intake trajectory. | Australian National Health and Medical Research Council (APP1046219 2013-17, APP144595 2018-22) |  |
| 32 | *Alkadi et al. 2024 (49)* | Mixed-effect Models, cross-sectional logistic regression model | A higher mean experience and prevalence (ECC) were observed in children with prolonged breastfeeding (≥24months) and high consumption of added sugars. | National Institute of Dental and Craniofacial Research NIDCR K23DE027412 and NIDCR R01DE031025 |  |
| 33 | *Mathias et al. 2024 (50)* | Effect measure modification (EMM) analysis was assessed by means of the relative excess risk due to interaction (RERI). Generalized linear models to estimate PR and RR | Sweet and non-sweet indices were positively  associated with the presence of cariogenic microorganisms, including *S. mutans* and *Candida*. | Brazilian Association of Collective Health (ABRASCO). Wellcome Trust (095582). National Council for Scientific and Technological Development (CNPq) and the Foundation for Research Support of the State of Rio Grande do Sul (FAPERGS). Pastoral da Criança. FAPERGS-PPSUS, Wellcome Trust (10735_Z_18_Z), and the Bernard van Leer Foundation (BRA-2018-178). Notice FAPERGS/CNPQ PRONEX 12/2014 (16.0471-4) and the CNPQ Universal Notice (454796/2014-5 and 426230/2018-3). Coordenação de Aperfeiçoamento de Pessoal de Nível Superiord Brasil (CAPES) Finance Code 001. |  |
| 34 | *Kerguen et al. 2025 (51)* | Logistic regression | Introduction of fruit juice or SSBs before 6 months of age was associated with increased ECC at 3.5 years | National Research Agency Investment for the Future program (ANR-11-EQPX-0038), French National Institute for Research in Public Health (IRESP TGIR 2009–01 program), Ministry of Higher Education and Research; Min- istry of Environment; Ministry of Health; French Agency for Public Health; Ministry of Culture, and National Family Allowance Fund. |  |
