# Supplementary material for: Measuring sugar intake in oral health birth cohort studies: a scoping review
Source: Front Nutr. 2026 Jan 7;12:1667487. doi: 10.3389/fnut.2025.1667487 (PMC12821232; doi:10.3389/fnut.2025.1667487)
Supplement: Supplementary file 4 [file Table_4.docx]

**Supplemental Table 4:** Covariates adjusted for in the statistical analysis

| **N°** | **Author/Year** | **Covariates adjusted for in the statistical analysis** | | | | |
| --- | --- | --- | --- | --- | --- | --- |
|  |  | ***Demographic factors*** | ***Socioeconomic factors*** | ***Behavioral*** | ***Clinical or interventional*** | ***Medications and others*** |
| 1 | *MacKeown et al. 2000 (23)* | - | - | - | - | - |
| 2 | *Habibian et al. 2001 (24)* | - | - | - | - | - |
| 3 | *Habibian et al. 2002 (25)* | - | - | - | - | - |
| 4 | *Warren et al. 2002 (26)* | - | - | - | - | - |
| 5 | *Marshall et al. 2003 (12)* | Child’s age at dental examination and child’s sex | - | Fluoride exposure* | - | - |
| 6 | *Öhlund et al. 2007 (27)* | - | - | - | Bacterial counts and oral hygiene scores | - |
| 7 | *Feldens et al. 2010 (28)* | - | - | - | - | - |
| 8 | *Tanaka et al. 2013 (29)* | Maternal age @baseline survey, child’s sex, child’s birthweight, age @first tooth eruption, age @oral examination | Family income, paternal and maternal educational levels | Maternal smoking during pregnancy, tooth-brushing frequency at fourth and fifth surveys, use of fluoride, household smoking at fifth survey, regular dental checkups | - | - |
| 9 | *Chaffee et al. 2015 (7)* | - | - | - | - | - |
| 10 | *Park et al. 2015 (30)* | Child’s sex, child’s birth weight, maternal age, maternal race/ethnicity, marital status, Prepregnancy BMI, parity @baseline | Maternal education, income-to-poverty ratio | breastfeeding duration, tooth brushing and sweet food intake at follow-up | - | - |
| 11 | *Wigen and Wang 2015 (31)* | Parental origin**, stability in family situation^, gender, age at dental examination | Maternal education | oral health behavior at 5 years of age^^ | - | - |
| 12 | *Peres et al. 2016 (11)* | Child’s sex, mother’s level of education | Family income | Breastfeeding, regularity of dental visit, toothbrushing habits | - | - |
| 13 | *Avasare et al. 2017 (32)* | Child’s age | Maternal education | Sports drinks, no night feeding practices, open cup | Presence of any decay, number of teeth more than the mean for entire group | - |
| 14 | *VanBuren et al. 2017 (33)* | - | - | - | - | - |
| 15 | *Feldens et al. 2018 (34)* | Child’s age, child’s sex, maternal age | Maternal education, household social class | Total carbohydrate intake | - | Allocation status in the nesting trial |
| 16 | *Bell et al. 2019 (35)* | Maternal age @birth, two parent household, weight status, child’s age @24HDR, gender, age introduced to solids | Index of Relative Socio-Economic Advantage and Disadvantage (IRSAD), household income, education attainment and work status prior to birth) | Breastfeeding duration | - | - |
| 17 | *Hu, Shijia et al. 2019 (36)* | Socio-demographic characteristics | | Oral hygiene habits | - | Perinatal and postnatal characteristics |
| 18 | *Bernabé et al. 2020 (8)* | Maternal age@delivery, child’s sex, child’s age (months), birthweight, | Maternal education, parental employment, level of deprivation of the area where the family lived | Smoking in pregnancy, breastfeeding and toothbrushing frequency | - | - |
| 19 | *Pitchika et al. 2020 (37)* | Child’s sex, age−/sex-standardized BMI categories | Parental education | Mode of SSD consumption | Plaque-affected sextants | Study cohort, energy content of SSDs and total energy intake |
| 20 | *Carvalho Silva et al. 2021 (38)* | Child’s sex, maternal age | Maternal education | Toothbrushing frequency, and eating before going to bed | - | - |
| 21 | *Feldens et al. 2021 (39)* | Maternal age @birth, gestational weight gain (kg), child’s sex, birth weight, birth height, child’s BMI z-scores @6months and @3years | Maternal education, family income | Sugar consumption index @6months, | @3 years: Developmental defects of enamel, visible plaque, previous dental visit, dental caries | @3years: Monthly sugar purchase (kg/person), Monthly oil purchase (kg/person), assignment group in the original trial (intervention or control) |
| 22 | *Manohar et al. 2021 (40)* | Child age, child gender, maternal age, maternal marital status, number of children in household | Maternal education, maternal work status, IRSAD | Sugary foods, breastfeeding duration, maternal smoking during pregnancy | - | - |
| 23 | *Marshall et al. 2021 (13)* | Child’s sex | Socioeconomic status | Other beverage intakes, fluoride intake, toothbrushing frequency | - | - |
| 24 | *Moreira et al. 2021 (41)* | Adolescent’s sex | Household income, adolescent educational level | Current smoking, and alcohol use | - | - |
| 25 | *Boustedt et al. 2022 (42)* | - | - | - | - | - |
| 26 | *Echeverria et al. 2022 (43)* | Maternal age in the perinatal period | Family income in the perinatal period and at 12, 24 and 48 mo, maternal education in the perinatal period and at 48 mo |  | whether the mother had received any oral health instruction from a health professional in the perinatal period and at 12, 24, and 48 mo | - |
| 27 | *Ha et al. 2022 (44)* | Child’s age and sex, maternal age, family composition | Maternal education, Area-level IRSAD | - | - | - |
| 28 | *Wu, Tong Tong et al. 2022 (45)* | - | - | - | - | - |
| 29 | *da Silva et al. 2023 (46)* | Maternal age, adolescent’s skin colour | Maternal schooling | Tooth brushing at least twice a day and always before bedtime at ages 5 and 10–­11 years | - | - |
| 30 | *Echeverria et al. 2023 (47)* | Maternal age in the perinatal period | Family income in the perinatal period, maternal education in the perinatal period | - | - | - |
| 31 | *Ha et al. 2023 (48)* | Child’s sex, age in month at the time of examination and birth weight | - | - | - | - |
| 32 | *Alkadi et al. 2024 (49)* | Infant sex, infant race, dad as care provider | Maternal education | Toothbrushing | - | History of antibiotics use |
| 33 | *Mathias et al. 2024 (50)* | Maternal age | Family income, mother’s level of education | - | Frequency of toothbrushing, child fed immediately before bed | - |
| 34 | *Kerguen et al. 2025 (51)* | Preterm birth, mother’s birth country. | Mother’s education level | Perceived maternal oral health | - | - |
| **Topical fluoride exposure to protect against caries was defined as the sum of fluoride from all water, other beverages, fluoride supplements, and dentifrices.*  **** *country of birth of the mother and father*  *^* *The stability of family status was measured by registering whether mother and father lived together or not in pregnancy and when the child was aged 5 years*  *^^tooth brushing frequency, fluoride lozenges, sugary drinks, sugary drinks at night*  *-SSD: sugar-sweetened drinks*  *-BMI: body mass index* | | | | | | |
